# Bridging the gap for diverse applications of parasites as advanced cancer therapeutics: current progress and future directions

**DOI:** 10.1186/s13027-025-00679-7

**Published:** 2025-07-29

**Authors:** Maha M. Eissa, Marwa H. El-Faham, Nahla El Skhawy

**Affiliations:** Department of Medical Parasitology, Faculty of Medicine, Alexandria University, Alexandria, Egypt

**Keywords:** Parasitic cancer therapeutics, Parasite immunotherapy, Cancer vaccines, Oncolytic agents, Immunomodulators, Gene therapy, Resistance to anti-cancer drugs

## Abstract

Cancer research is constantly evolving to yield successful innovations. A plethora of pre-clinical studies have illustrated the promising potential utility of parasites and parasite-derived molecules in cancer therapy. In this review, we underscore, for the first time, the possible multifaceted applications of parasites in the field of oncology, aiming to draw attention to the vital role of parasite-derived cancer therapy and offer novel insights for the evolution of advanced cancer therapeutics. Several studies have demonstrated that parasites offer a variety of strategies for cancer therapy. These include acting as immunotherapeutics such as cancer vaccines, therapeutic antibodies, adjuvants, immunomodulators, oncolytic agents, and NF-κB inhibitors. Additionally, they can be utilized in targeted therapy, gene therapy, and in combination with current cancer treatments to synergistically enhance their effectiveness. A notable strategy is parasites’ ability to overcome tumor resistance to chemotherapy, a significant obstacle in cancer therapy. There is still much to explore about parasite-based anti-cancer therapies. With further research and the translation of parasitological discoveries into effective cancer interventions, parasites may hold the key to effectively treat cancer in the near future.

## Introduction

Cancer is a group of chronic and progressive disorders where abnormal cells grow uncontrollably [1]. It poses a significant worldwide socioeconomic challenge, resulting in millions of additional cases and fatalities annually [2]. The number of cancer deaths has risen by 40% in a little over a decade, and it is projected that by 2050, there will be over 35 million additional cases, a 77% increase compared to 2022 [3]. Factors like genetic abnormalities, inflammation, poor diet, exposure to radiation, and toxin intake have been associated with cancer development and progression [4].

Cancer is one of those persistent diseases requiring speedy policies with targeted applications. Consequently, it has gained a lot of attention and focus from researchers toward investing in policies for cancer therapeutics. Effective cancer treatment is still a major challenge for many patients [5]. Commonly used traditional treatments include surgery, chemotherapy, radiotherapy, and hormonal treatment. Newer cancer treatments include the development of cancer vaccines, therapeutic antibodies, immune system modulators, and gene therapy drugs. Despite advancements in cancer treatment through personalized medicine and immunotherapy, the global incidence of cancer is rising [1]. Additionally, cancer therapy resistance is a major concern and poses a significant hurdle to treatment [6]. Therefore, continuous research is essential for the development of more efficient and innovative cancer treatments [7].

Evidence has shown a link between some pathogenic infections and the occurrence of cancer, such as *Schistosoma haematobium* and Human papillomavirus. However, a historical detour took place, and some pathogens, including viruses, bacteria, and parasites, demonstrated remarkable antineoplastic activity. For example, Hepatitis B virus vaccine has shown prophylactic efficacy against hepatocellular carcinoma [8]. Modified forms of certain bacteria as attenuated *Mycobacterium bovis* (bacillus Calmette-Guérin-BCG), displayed potent therapeutic activity against urinary bladder carcinoma and metastatic melanoma [9, 10]. This ensured their promotion to clinical trials and has now received credential approval as prophylactic and therapeutic strategies for these types of cancers. On the other hand, a wide variety of parasites have been investigated for their antineoplastic potency against a wide array of cancer types. Fluctuating between live parasitic infection, attenuated parasites, parasitic antigens, proteins, antibodies, and genes, reports are continuously arising, justifying and fortifying the hypothesis, thus establishing a concrete platform for the use of parasites as antineoplastic agents. These studies involved a broad range of cancer cells, parasites, and their derivatives conducted in vitro and in vivo. Not only helminths, but protozoa have also gained a lot of attention and have been frequently investigated [11, 12].

Despite the massive amount of available data, there is a lack of a clear policy for potential prospective and future applications of this data. Upgrading experimental work to a clinically applicable tool is the ultimate long-term goal of research and a qualitative shift towards the coronation of tons of experimental work. This is not a straightforward path and usually encloses struggles, obstacles, and experimental trials that enfold defeats and victories. Therefore, establishing a plan and sketching guidelines for promising applications can raise enthusiasm toward a more precise analysis and practical implementation of parasites for cancer-targeted therapy. Hence, this review outlines the potential diverse applications of parasites in oncology, aiming to draw attention to the vital role that parasite-derived cancer treatment has made. Thus, providing novel insights for the evolution of advanced cancer therapeutics for incurable cancer.

## Applications of parasites and Parasite-Derived molecules in Cancer treatment

**Fig. 1:**
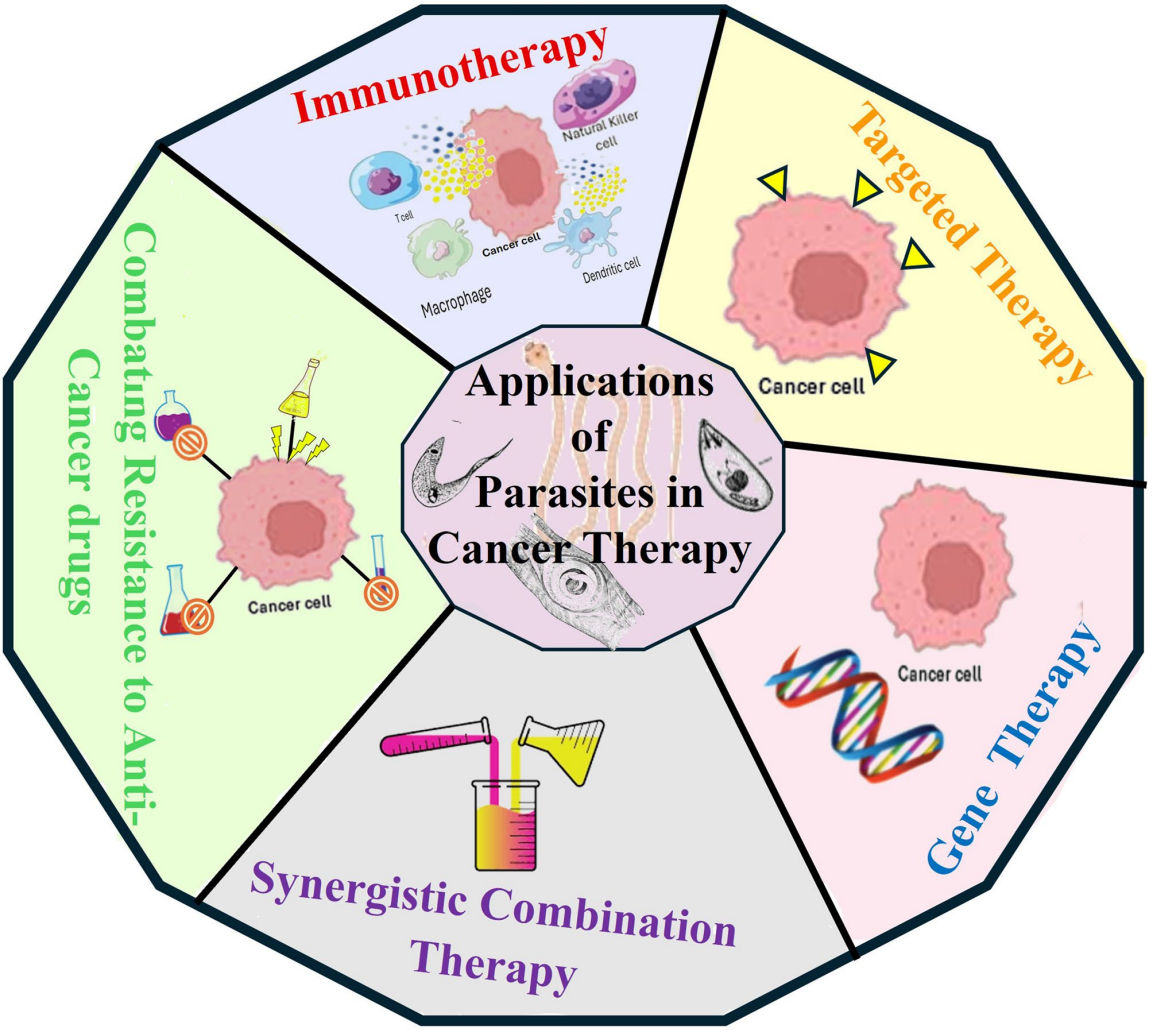
Representation of various applications of parasites for cancer therapy

Research has demonstrated that parasites and their derived molecules have diverse applications in oncology. These findings offer new possibilities for developing innovative cancer therapies, such as immunotherapies, targeted therapy, gene therapy, synergistic combination therapy, and combating drug resistance, as shown in Fig. 1.

### Immunotherapy

The dominance of an immunosuppressive state in cancer underscores the importance of cancer immunotherapy. This type of treatment encompasses various approaches that activate the immune system to identify and eliminate cancerous cells. These drugs are specifically delineated to alert the immune system against cancer cells that have undergone mutations to avoid detection by the immune system. Immunotherapy can be achieved by either manipulating the patient’s immune cells using internal resources or by stimulating the immune system through external resources [13].

**Fig. 2:**
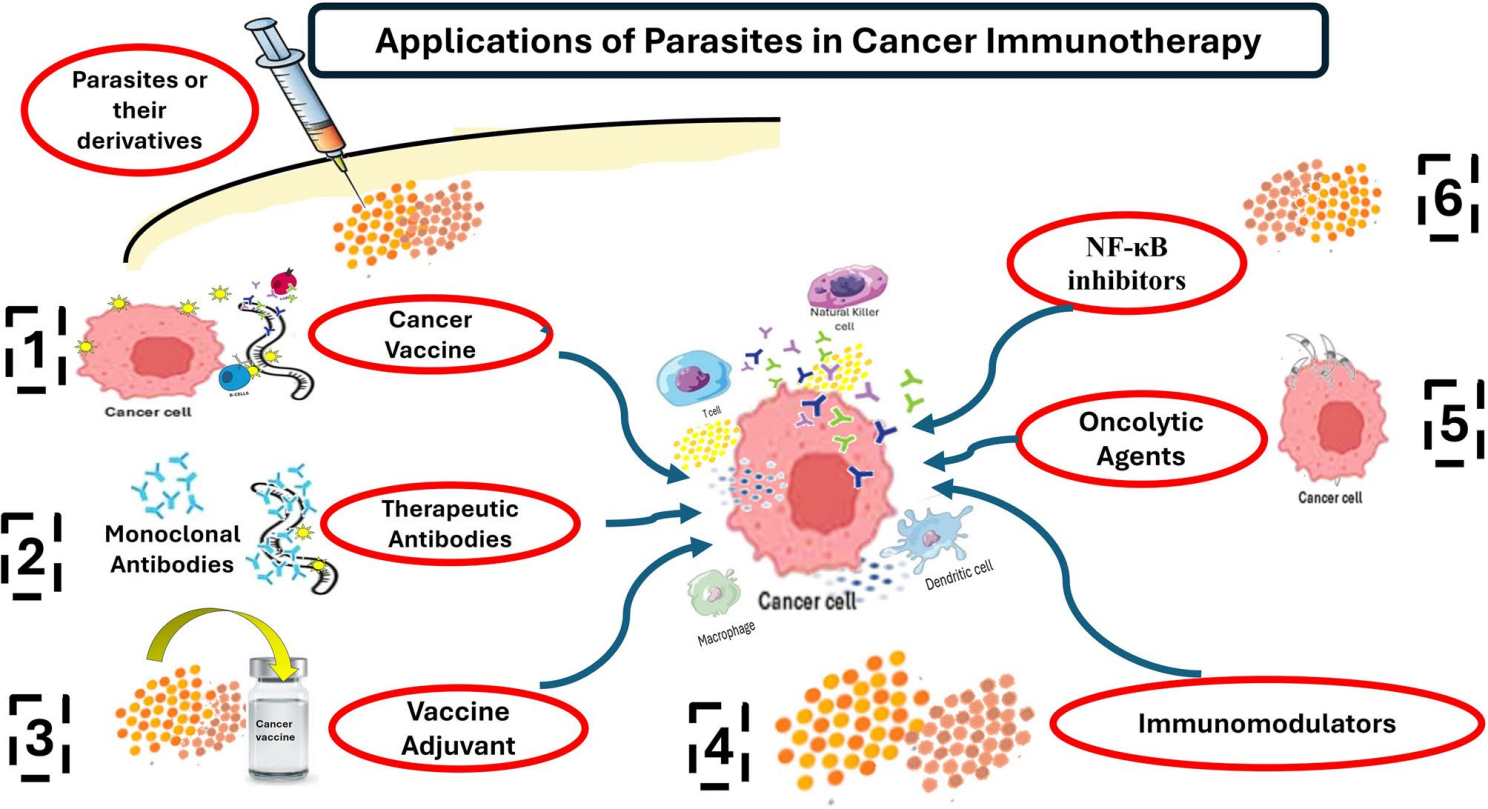
Diagrammatic illustration of the applications of parasites in cancer immunotherapy

Cancer immunotherapy encompasses various categories, each with distinct mechanisms and risks. The selection of immunotherapy depends on the cancer type and stage. Options include Chimeric antigen receptor T-cell therapy (CAR-Tcell), a form of adoptive T-cell transfer, cancer vaccines, monoclonal/polyclonal or therapeutic antibodies, oncolytic virus therapies, immune-system modulators, cytokine therapies, and immune checkpoint inhibitors which are negative regulators of T cells [14], as shown in Fig. 2.

#### Cancer vaccine candidates, and therapeutic antibodies

Cancer vaccines have a fundamental role in cancer immunotherapy [8, 13]. One of the main obstacles in developing efficient cancer vaccines with tumor-associated antigens is their limited ability to overcome immune tolerance. In contrast, the immune system can detect pathogens more easily than cancerous cells because they are foreign, resulting in a more robust immune response [15].

The hygiene hypothesis suggests that reduced exposure to microorganisms during childhood may be associated with immune-related disorders like allergic and autoimmune conditions [16–18]. In addition to several epidemiological studies that support this hypothesis [19–21], a plethora of experimental studies have contributed to the progression of the hygiene hypothesis from an observational theory to an experimental theory. Microbes play a crucial role in supporting the development of a normal immune system. Early exposure to a variety of microbes helps educate the immune system, fostering tolerance to allergens and decreasing the risk of improper immune responses. Current research supports the potential for manipulation of helminthic parasites and their derivatives to treat and even prevent immune dysregulation in the form of atopic diseases and other immune-mediated disorders, such as allergic diseases and fatal anaphylaxis [22–25]. There are also experimental studies suggesting the ability of helminths to ameliorate symptoms of autoimmune diseases such as TNBS-induced colitis, arthritis, and psoriasis [26–30]. This theory has been expanded to include other disease areas, such as psychiatric disorders and cancer as a potential outcome, leading to the consideration of utilizing pathogens and their components in cancer immunotherapy [17]. Examples include the use of the BCG vaccine for treating urinary bladder cancer [9] and skin cancer like melanoma [10], as well as human papillomavirus and hepatitis B virus vaccines, as prophylactic vaccines against cervical cancer and hepatocellular carcinoma, respectively [8].

An alternative method to boost antitumor immune responses involves utilizing monoclonal antibody-based strategies (mAb) to target immune cells rather than tumor antigens. These laboratory-produced products mimic the body’s innate immune response to fight cancer. Monoclonal antibody therapy has become a key element in cancer treatment, complementing surgery, chemotherapy, and radiation. These antibodies specifically target and eliminate cancer cells without harming healthy cells. They have various mechanisms of action, including flagging cancer cells, destroying cell membranes, inhibiting cell proliferation, clogging immune system inhibitors, directly assailing cancer cells, delivering chemotherapy and radiation, tying to immune and cancer cells, and preventing blood vessel growth. They can also stimulate long-lasting antitumor immune responses. This therapy is used for treating leukemia, lymphoma, and solid tumors [31].

Several research studies have demonstrated that there are common antigenic epitopes shared between some parasites and various types of cancer, supporting the molecular mimicry theory proposed in the 1960s [32]. Fundamental cancer markers for cell invasion and metastasis like Tn, Tk, sial Tn, and TF, which are early glycosylation products of serine and threonine, are masked by the full glycosylation chains [10, 33–35]. These markers are considered specific to cancer [34]. Interestingly, these cancer-associated mucin-type O-glycans are also found in specific helminths like *Echinococcus granulosus* (*E. granulosus*), *Fasciola hepatica* (*F. hepatica*), adult *Schistosoma mansoni* (*S. mansoni*) and its schistosomula stage [15]. This shared antigenicity suggests a potential new direction for cancer biotherapy using parasites and their antigens for the evolution of highly immunogenic parasite-derived cancer vaccine candidates for prophylactic and therapeutic purposes.

Interestingly, in a murine model of colon cancer, Eissa et al.., 2019 found that *S. mansoni* cercarial antigen and *Trichinella spiralis* (*T.spiralis*) larval antigen have significant immunomodulatory effects. This led to an increase in serum IL-10, splenic CD4 + T-cells, and intestinal FoxP3 + Treg cells, as well as a decrease in serum IL-17 [36]. However, only the *S. mansoni* cercarial antigen showed anti-colon cancer activity, resulting in reduced tumor size and neoplasia number per mouse [37]. This selective antineoplastic activity of *Schistosoma* antigen against colon cancer was attributed to shared antigens between *S. mansoni* and colon cancer cells as both have been shown to express Tn antigen [38, 39].

Therefore, studying shared antigens in parasites and cancerous cells could lead to the evolution of vaccines that efficiently activate the immune system to attack and eliminate cancer cells, similar to how it responds to parasites. These vaccines may also have the potential to target infectious and autoimmune diseases, addressing multiple health concerns.

Additional research and escalating promising results to clinical studies are needed to evaluate their safety and efficacy in these fields.

##### Parasites sharing antigenic epitopes with different types of cancer

Parasites sharing antigenic epitopes with various types of cancer are presented in Table 1.

**Table 1:** Parasites sharing antigenic epitopes with different types of cancer

Parasite	Cancer type	Shared antigen	Reference
***E. granulosus*** **(hydatid cyst)**	Breast cancer	Protein bands (~ 27/28 kDa)	[33, 41]
	Colorectal cancer	Colon cancer cells (CT26) mortalin exhibited 60% similarity with *E. granulosus* HSP-70	[46]
	Lung cancer	Antibodies specific to hydatid cyst fluid recognized intracellular and membrane molecules in lung cancer cells	[49]
	Bone tumors	Two antigens, 70 kDa, and 43 kDa	[52]
***T. spiralis***	Myeloma	Tropomyosin	[60]
		*T. spiralis* gene encoding TS2 protein	[61]
		*T. spiralis* genes encoding NKTR, RpL41, ANXA2, and Rbbp4 proteins	[54]
	Lung cancer	T.sHSP-DQ 986457)	[62]
		Two protein bands (~ 70 kDa and 35 kDa )	[64]
	Osteosarcoma	Gene tumor protein -D52 (TPD52)	[59]
	Breast cancer	Two protein bands (~ 70 kDa and 35 kDa )	[64]
***S. mansoni***	Lung cancer	One protein band (~ 80 kDa )	[64]
	Breast cancer	One protein band (~ 80 kDa )	[64]
***T. cruzi***	Neuroblastoma	Antibodies to *T. cruzi* reacted with antigenic proteins in neuroblastoma cells	[77]
	Colorectal cancer	Sialyl-Tn antigen	[74]
	Breast cancer	anti-*T. cruzi* antibodies cross-reacted with breast cancer cell lines	[74]
	Acute lymphocytic leukemia	Rabbit anti-*T. cruzi* polyclonal antibodies reacted with acute lymphoblastic leukemia cells protein extract	[77]
		100 kDa, nucleolin	[80]
	Lung cancer	Antibodies to *T. cruzi* identified intracellular and membrane molecules in lung cancer cells.	[76]
***S.equina***	Hepatocellular carcinoma	75 kDa	[81]
	Breast cancer	Two protein bands, 70 kDa and 75 kDa, corresponding to HSP70 and G6PD, respectively	[81]
***T.gondii***	Breast cancer	Human and rabbit anti-*T. gondii* antisera strongly reacted with 4T1 and MCF7 breast cancer cells	[93, 94]
	Melanoma	The mouse melanoma cells strongly reacted with anti-*T. gondii* antiserum	[94]

***Echinococcus granulosus*** **(*E***. ***granulosus*)**

Several experimental studies have demonstrated shared antigenicity and antineoplastic activity of the larval stage of the dog tapeworm, *E. granulosus* with breast [37, 40–45], colorectal cancer (CRC) [46–49] and lung cancers [49–51]. Additionally, it has been shown to have antigenic similarity with bone cancers [52].

Antisera from rabbits immunized with hydatid cyst antigens showed reactivity with breast cancer cells (4T1) [41, 45] and hydatid cyst wall antigens exhibited cross-reactivity with the sera of breast cancer patients at a distinct band (~ 27/28 kDa) [33, 41]. Prophylactic immunization with this band in a mouse model of breast cancer extended their survival time, inhibited breast tumor growth, and reduced metastasis by increasing levels of tumor necrosis factor-alpha (TNF-α), interleukin-2 ( IL-2), IL-4, and interferon-gamma (IFN-γ) [41, 53]. Mass spectrometry analysis revealed proteins with high homology to cancer cell antigens in the ~ 27/28 kDa band, leading to an antitumor immune response [41].

Another study found that colon cancer cells (CT26) mortalin exhibited a 60% similarity with *E. granulosus* heat shock protein 70 *(* HSP-70) [46], potentially explaining the antineoplastic effects of *E. granulosus* on colorectal cancer in a mouse model [46, 48].

Additionally, the immunoelectrophoretic analysis showed a broad band when serum from a patient with lung carcinoma was examined with fluid from hydatid cyst [51]. Flow cytometry analysis showed that antibodies specific to hydatid cyst fluid were able to recognize lung cancer cells’ membrane and intracellular molecules [49], which supports its antitumor effect as demonstrated by the in vitro potential of the serum from patients with hydatid disease to cause a cytotoxic effect on NCI-H209/An1 human small-cell lung cancer cells [50]. Furthermore, antigens from hydatid cysts reacted with serum from patients with bone cancer, identifying distinct antigens at 70 kDa and 43 kDa [52].

***Trichinella spiralis (T. spiralis)***

Research has shown that *T. spiralis*, a relatively small intestinal nematode worm, exhibits similar antigenicity and potential antineoplastic activity against myeloma [54], lung cancer [55–58], and osteosarcoma [59]. Antigens associated with myeloma have been identified in the helminth parasite, *T. spiralis*, leading to immune responses that cross-react between antigens of the myeloma cell SP2/0 and positive sera for *T. spiralis*, as well as of *T. spiralis* antigens and positive sera for myeloma cell SP2/0. Tropomyosin, an acidic protein present in *T. spiralis* myofibrils, has been identified as a significant component of myeloma-associated antigens located on its surface. Immunization with tropomyosin or crude *T. spiralis* antigen led to an anti-tumor response by increasing CD19^+^ B lymphocytes, and CD8^+^, CD4^+^T lymphocytes [60].

Further studies identified novel antigen genes in *T.spiralis* responsible for its antitumor activity, such as TS2, which showed cross-reactivity with Sp2/0 myeloma cells. Sequencing analysis identified a 569-base pair (bp) fragment that encodes 136 amino acids. The gene includes 4 potential N-Arg dibasic convertase cleavage sites, one glycosaminoglycan attachment site, and one peptide C-terminal amidation site. Bioinformatic analysis predicted six antibody binding sites [61]. Furthermore, genes that encode NKTR, RpL41, ANXA2, and Rbbp4 were abundant in tumor cells from *T. spiralis*-infected mice, indicating their potential role in the antitumor activity of *T. spiralis* [54].

A small heat shock protein (sHSP-DQ 986457) was identified as a shared gene between *T. spiralis* and Lewis lung cancer (LLC) cells. Western blotting analysis showed that recombinant sHSP reacted with LLC cell antiserum, indicating an association between the two [62]. Moreover, immunization with *T. spiralis* sHSP suppressed tumor growth in LLC murine model through stimulation of mucosal, cellular, and humoral immunity [63].

The Tumor protein D52 (TPD52) antigen gene was identified as a cross-antigen in *T.spiralis* and human osteosarcoma, exhibiting in vitro and in vivo anti-osteosarcoma activity. Anti-TPD52 antiserum effectively suppressed the growth of MG-63 human osteosarcoma cells and triggered apoptosis in a concentration-dependent manner. In a mouse osteosarcoma model, the anti-TPD52 antiserum enhanced immunity more effectively than the anti-*T. spiralis* antiserum, leading to increased serum levels of TNF-α, IL-12, and IFN-γ, without inducing histopathological changes. This resulted in significant inhibition of osteosarcoma growth by boosting immunity and inducing apoptosis [59]. Additionally, a recent study conducted by Eissa et al., in 2025 showed cross-reactive antigens between *T. spiralis* and human MCF-7 breast and A549 lung cancer cell extracts using rabbit polyclonal anti-serum against *T. spiralis.* These antigens were observed at molecular weights of approximately 70 and 35 kDa using SDS-PAGE and immunoblotting techniques [64].

***Schistosoma mansoni***

*Schistosoma* is a helminthic parasite causing schistosomiasis, a neglected tropical disease. It is a paradoxical parasite as one species, *Schistosoma haematobium* (*S. haematobium*), is classified as a group I biological carcinogen [65], while another species, *Schistosoma mansoni* (*S. mansoni*), has shown anti-neoplastic activity in pre-clinical studies against sarcoma [66], colon [36], CRC [67], and breast cancers [68]. A recent study in 2025 demonstrated the existence of shared antigens between the *S. mansoni* parasite and human A549 lung and MCF-7 breast cancer cells, with a molecular weight of around 80 kDa through SDS-PAGE and immunoblotting [64].

***Trypanosoma cruzi (T.cruzi)***

*T.cruzi*, is a protozoan parasite responsible for Chagas disease, also called American trypanosomiasis. Experimental studies have demonstrated that *T. cruzi* demonstrated similarity and antineoplastic activity against breast [69–75], CRC [72, 74], and lung cancers [76]. It has also been found to share antigens with neuroblastoma and acute lymphoblastic leukemia [77].

Antibodies against *T.cruzi* developed in experimental animals vaccinated with *T. cruzi* antigens cross-reacted in vitro with colon and breast cancer cells [74] as well as with intracellular and membranous molecules in lung cancer [76]. These findings suggest the existence of shared antigens that can trigger antibody-dependent cellular cytotoxicity [74]. One such antigen is the mucin-type cancer-associated sialyl-Tn antigen found in both *T. cruzi* and colon cancer cells [78]. potentially explaining the lack of colorectal cancer in chagasic megacolon patients [79].

Furthermore, *T. cruzi* antigenic proteins were found to be shared with neuroblastoma and acute lymphoblastic leukemia [77]. Anti- *T. cruzi* antibodies reacted with acute lymphoblastic leukemia and neuroblastoma cells, indicating a cross-reactive immune response. These antibodies identified 35.5% of the surface antigens present on B cells from the acute lymphoblastic leukemia SUPB15 and exhibited complement-dependent cytotoxicity. Mass spectrometry identified nucleolin at 100 kDa as a potential protein involved in *T.cruzi ’s* antitumor effect [80].

***Setaria equina (S. equina)***

*S. equina* is a type of filarial parasite frequently located in the equines’ peritoneal cavities worldwide. Studies have shown that *S.equina* shares antigenicity with breast cancer and hepatocellular carcinoma (HCC) [81]. Western blotting showed similar protein bands at 75 and 70 kDa in *S. equina* adult worms, MCF-7 human breast cancer, and Huh-7 hepatoma cells, possibly corresponding to Glucose-6-phosphate dehydrogenase (G6PD), and HSP-70 respectively [81]. This was identified through western blotting and indirect ELISA using anti-*S. equina* extract raised in rabbits and anti-diethylcarbamazine citrate polyclonal IgG antibodies. The shared antigens between *S.equina* and HCC may explain the antineoplastic effect of *S.equina* excretory-secretory products on HCC in a rat model [82, 83].

***Toxoplasma gondii (T. gondii)***

Several experimental studies have demonstrated the inhibitory effect of *T. gondii* on breast cancer [84–87] and melanoma growth [88–92]. Furthermore, rabbit anti-*T. gondii* antisera effectively killed the breast cancer 4T1 cell line in vitro [42]. Flow cytometry analysis showed that both human and rabbit anti-*T. gondii* antisera strongly reacted with breast cancer cells (MCF7 and 4T1) [93, 94], indicating potentially shared epitopes between *Toxoplasma* antigens and breast cancer cells.

A study investigated how various antibodies, such as those targeting *T. gondii*, *Trichomonas vaginalis* (*T.vaginalis*), protoscolices antigens, and hydatid cyst fluid, interacted with mouse melanoma cells through flow cytometry analysis. Interestingly, only *T. gondii* antiserum showed strong reactivity with mouse melanoma cells, indicating potential common antigens between melanoma and *Toxoplasma* that could boost antitumor immunity. Meanwhile, antibodies against hydatid cyst fluid, *T. vaginalis*, and protoscolices antigens did not exhibit strong reactivity with mouse melanoma cells [94].

Therefore, additional research is urgently needed to investigate, identify, and assess shared antigens between parasites and various cancer types. This could lead to the development of cancer vaccine candidates derived from parasites that are highly immunogenic for use in cancer immunotherapy. Furthermore, the potential development of monoclonal and polyclonal antibodies against these shared antigens could offer a novel approach for targeted cancer immunotherapy. Moreover, the versatile nature of these antibodies as a treatment option could pave the way for new and creative approaches for treating cancer with significant implications for cancer care.

#### Vaccine adjuvants

Vaccine adjuvants are essential for boosting the immune response to vaccines. They are substances used in vaccine design to enhance immune responses. They are classified into two categories: immunostimulants and delivery systems. Immunostimulants like Toll-like receptor (TLR) agonists, damage-associated molecular patterns, and pathogen-associated molecular patterns (PAMPs), will trigger antigen-presenting cells (APCs) to boost antigen presentation and co-stimulatory signaling leading to adaptive immune enhancement. Delivery systems such as poly(lactide-*co*-glycolide), lipid nanoparticles, and caged protein nanoparticles improve the uptake and presentation of antigens by APCs. Approved adjuvants include aluminum, emulsion-based adjuvants, TLR agonist molecules, and particulate adjuvants [95]. Investigational platforms include cyclic dinucleotides [96], manganese adjuvants [97], new water-in-oil mini-emulsions [98], polymer particles [99], and inorganic nanomaterials [100], among others.

In recent years, parasites have demonstrated potent immune activation potential, making them valuable adjuvants for cancer vaccine immunotherapy. Several studies have highlighted the adjuvant properties of parasites and their derived molecules to boost the immune response toward tumor-associated antigen vaccines through acting either as delivery systems or immunostimulants. For instance, *Plasmodium yoelii*, genetically attenuated *Plasmodium* sporozoites, and a non-pathogenic recombinant *T. cruzi* clone have shown promising results as delivery system adjuvants.

*Plasmodium yoelii*, a species of rodent *Plasmodium*, is commonly used to simulate malaria infection in laboratory mice. It has been verified as a vaccine carrier for immunotherapy of hepatocellular carcinoma (HCC). The *P. yoelii* 17XNL strain which expresses murine glypican-3 protein (*P*.*y*-GPC3); that is highly expressed in Hepa1-6 cells, successfully suppressed tumor growth, and triggered a strong GPC3-specific cytotoxic T lymphocyte (CTL) immune response. It also raised levels of Th1-associated cytokines such as IFN-γ, IL-2, and TNF-α and enhanced the growth of the CD8α + dendritic cell (DC), which exhibited higher expression of CD86 and CD80 molecules [101]. Additionally, infection with *P. yoelii* alone suppressed HCC recurrence and metastasis, leading to improved prognosis in orthotopic HCC murine models. This beneficial effect was achieved by suppressing CC-chemokine receptor 10-mediated PI3K/Akt/GSK-3β/Snail signaling and preventing epithelial-mesenchymal transition. These findings indicate a potential new approach for the treatment of HCC recurrence and metastasis [102].

Similarly, the use of attenuated *Plasmodium* sporozoites as a carrier for MAGE-A3, a lung cancer-specific antigen, triggered robust MAGE-A3- specific CD8^+^ T cell responses, leading to improved mice survival rates, and suppression of tumor growth [103]. Notably, immunization with these genetically attenuated *Plasmodium* sporozoites alone stimulated innate immunity, and suppressed angiogenesis, and lung cancer growth [104]. Additionally, combining *Plasmodium* infection with a DNA lung cancer vaccine (pcDNA3.1-hMUC1) enhanced the immune response and synergistically affected the tumor [105].

Additionally, a non-pathogenic recombinant *T. cruzi* clone was used as a vaccine carrier to express NY-ESO-1, a cancer-testis antigen. This approach induced robust and prolonged T cell-mediated immunity, providing effective protection against melanoma in the mouse model [106]. When the blockade of cytotoxic T Lymphocyte Antigen 4 was combined with it, a significant response was observed. This combination boosted the number of NY-ESO-1-specific effector CD8^+^ T cells that produced IFN-γ and facilitated lymphocyte migration to the site of the tumor, ultimately controlling established melanoma in mice [107].

On the other hand, parasites’ derived molecules have shown potential as immunostimulant adjuvants. Research has shown that molecules derived from *T. cruzi*, like calreticulin, and *T.gondii*, like profilin-like protein and dense granule protein 6 (GRA6Nt) can boost the immune response to tumor-associated antigen vaccines. For example, in a mouse model of B16-F10 melanoma, vaccination with a plasmid encoding survivin, a tumor-associated antigen, combined with *T. cruzi* recombinant calreticulin (r*Tc*CRT), resulted in synergistic suppression of tumor growth and the stimulation of humoral anti-r*Tc*CRT immunity [108]. Furthermore, Toll-like receptor agonists derived from *T. cruzi*, such as CpGs oligodeoxynucleotides and glycolinositolphospholipids, have been found to be effective adjuvants. These agonists have demonstrated the ability to generate protective immunity and slow down the progression of B16F10 melanoma cells that express the cancer-testis antigen (NY-ESO- 1) in a mouse model. Additionally, these agonists promote CD4 + T cells to release IFN-γ. It is worth noting that CpGs oligodeoxynucleotides from the parasite, rather than glycoinositolphospholipids, triggered a robust IFN-γ reaction from CD8^+^ T lymphocytes [109].

Furthermore, profilin-like protein from *T. gondii* (*Tg*PLP) functions as a Toll-like receptor (TLR) agonist and has the potential to boost antitumor immune responses when employed as a vaccine adjuvant. In a colorectal carcinoma murine model, recombinant *Tg*PLP was shown to improve survival and reduce the size of CT26 tumor in mice immunized with an autologous whole-tumor cell vaccine (AWV). *Tg*PLP treatment also led to an increase in the expression of antigen-presenting cell markers such as major histocompatibility MHC class I and II), as well as B7.2, and B7.1, along with cytokines like IFN-γ and IL-12 in mice. Mice that received vaccinated with both *Tg*PLP and AWV showed elevated levels of immune cells (CD8^+^ and CD4^+^T cells, macrophages, and natural killer (NK) cells in the spleen as well as higher total IgG and IgG2a levels in comparison to those immunized with AWV alone [110].

Likewise, the amino-terminus region of *T.gondii* dense granule protein 6 (GRA6Nt) also triggers cancer-specific immunity. In a murine model of MC38 CRC, the recombinant GRA6Nt (rGRA6Nt) protein serves as an efficient adjuvant when combined with non-replicable colorectal cancer MC38 cells treated with ionizing radiation or mitomycin C. This strategy enhances protective immunity against challenges with MC38 tumor cells, resulting in decreased tumor growth and increased CD8^+^ T cells in the tumor. CD8^+^ T cells display heightened activity, producing IFN-γ and granzyme B when exposed to cancer cells in vitro. This immunization is specific to CRC MC38 cells and has minimal impact on EL4 lymphoma tumors [111].

Therefore, the exploration of parasitic organisms to create a novel parasitic adjuvant platform for designing various cancer vaccines is a promising avenue to pursue and warrants further investigation.

#### Immune-system modulators (Immunomodulators)

Immunomodulators are used to enhance the body’s immune response. They can target specific parts of the immune system or have a broader effect on the entire body. In addition to immunomodulatory agents being tested in cancer immunotherapy trials, pathogens, and their products are emerging as promising candidates for cancer immunotherapy [15, 112]. The concept of using pathogens to modulate the immune system has been accepted and applied in oncology for some time. Viruses and bacteria are among the pathogens explored for their potential in cancer immunotherapy. Additionally, parasites have an immunomodulatory effect that triggers a noticeable immune response in the host when exposed to the parasite or its antigens. This effect can be beneficial and utilized to benefit the host. Research has explored this immunomodulatory effect in various medical fields, including cancer, to counteract the immunosuppressive dominance seen in cancer. Recent evidence suggests potential benefits in autoimmune diseases such as arthritis [28], bronchial asthma, inflammatory bowel diseases, multiple sclerosis [113], and cancer [36].

Parasites can have a negative impact on cancer cells by modulating the immune system and promoting antineoplastic activity. For example, *Plasmodium* infection can alter immune responses by releasing cytokines, activating specific immune cells, and altering macrophage function. Research has shown that infection with *Plasmodium* can trigger an immune response that inhibits cancer growth by releasing TNF-α and IFN-γ, activating natural killer (NK) cells, promoting the proliferation of tumor-specific T-cell proliferation, enhancing CD8^+^ T cell activity, and inhibiting angiogenesis [105]. While using live parasites for cancer treatment is impractical due to long-term infection risks, parasitic-derived molecules showed promise in boosting the immune system without causing infection. These molecules could potentially harness the antitumor immunomodulatory benefits of parasites [9, 112].

Helminths and protozoa-derived molecules have shown promising antineoplastic and immunomodulatory properties. For instance, mucin-type O-glycans derived from *E. granulosus* have demonstrated antitumor effects by activating a pro-inflammatory Th-1 immune response. In another study, the synthetic peptide GK1 from cysticerci of *T. crassiceps* exhibited anti-melanoma activity in B16-F10 melanoma murine model, leading to prolonged mice survival, slowed tumor growth, and increased tumor necrosis. GK1 enhanced the immune response by stimulating pro-inflammatory cytokines [114].

Additionally, *T.spiralis* retinoblastoma binding protein 4 and c-Ski proteins among others have shown potential antitumor effects [54, 115]. During infection with *T.spiralis*, retinoblastoma binding protein 4, a potent regulator of cellular proliferation and chromatin structure is observed in myeloma cells. This protein has a role in inhibiting breast cancer MCF 7 cells [116]. Similarly, the oncoprotein, c-Ski, expressed in muscle cells infected with *Trichinella*, inhibits tumor growth by producing inhibitory cytokines like tumor growth factor beta (TGF-β) [115, 117]. Furthermore, infection with *T. spiralis* inhibited tumor growth and reduced lung metastasis of B16-F10 melanoma cells by decreasing the production of certain chemokines, including IL-4, CXCL9, CXCL1, CXCL10, and CXCL13 [118]. Therefore, it is noteworthy to investigate other immune-regulatory molecules from *T. spiralis* such as TGF-β ligand homolog, cystatin, cathepsin B-like protein, glutathione-S-transferase, calreticulin and 7C2C5Ag glycoproteins (45, 49 and 53-kDa proteins) for their potential antineoplastic activity [119–123].

Protozoan parasite-derived molecules have been shown to overcome the tumor immunosuppressive microenvironment. Parasites like *T. gondii* and *T. cruzi* show promise in experimental trials for reactivating the immune response, overcoming the tumor’s immunosuppressive microenvironment, inhibiting angiogenesis, and inducing apoptosis, making them potential candidates for cancer immunotherapy [112]. GRA15II, a virulence-associated protein found in the dense granule protein of *T. gondii*, induces macrophage polarization and restricts tumor growth in the HCC mouse model, by modulating cytokine profiles. This molecule shows promise in enhancing host innate immunity against cancer [124]. Profilin, another *T.gondii-*derived molecule as well as whole *T. gondii* lysate show promise in treating pancreatic cancer in a murine model. It induced antitumor activity by enhancing the infiltration with CD8 + and CD4 + T cells into tumors [125]. *T.gondii* lysate also activates innate immunity in mice with colorectal carcinoma CT26 tumor, resulting in decreased tumor growth and levels of the metastatic marker TIMP-1 [126]. Additionally, non-replicating *T. gondii* uracil auxotrophs secrete effector proteins that trigger antitumor immune response in mice by activating host immune responses through CD8α^+^ DC, the IL-12/IFN-γ Th 1 pathway, and CD8^+^ and CD4^+^ T cells, thereby reversing immune suppression in the ovarian tumor microenvironment [127].

Calreticulin (*Tc*CRT), P21, and J18 from *T. cruzi* are antitumor proteins. *Tc*CRT is released into the extracellular space to modulate the immune response. It contains an N-terminal vasostatin-like domain that can attach to endothelial cells and inhibit angiogenesis. Studies have shown that r*Tc*CRT has antiangiogenic and antitumor effects, in mammary (TA3 MTXR) [69, 71] and melanoma (B16-F10) murine models [108]. Recombinant *T. cruzi* P21 has diverse biological properties including immune cell chemotaxis, attaching to CXCR4 receptors in macrophages, and inhibiting angiogenesis. Research has demonstrated that *T. cruzi* rP21 attaches to CXCR4 receptors on triple-negative breast cancer cells, leading to receptor internalization and reducing their migration, invasion, and progression [82]. In a separate study, treating melanoma-bearing C57BL/6 mice with a recombinant protein called J18,which is based on the *T. cruzi* surface molecule GP82 fused with glutathione-S-transferase (GST), resulted in an antitumor response leading to smaller tumors and increased mice survival [128].

Other parasitic-derived molecules such as *Leishmania* sphingolipid-1(LSPL-1) and *Eimeria species* (*E.*) soluble protein have been shown to induce immunostimulatory effects. In a murine model of B16F10 cell-induced xenograft melanoma, LSPL-1 triggered an immune response, improved mice survival, and decreased tumor size in a dose-dependent manner. Additionally, it decreased angiogenic factors like vascular endothelial growth factor (VEGF), CD34, and Ang-2, without harming the murine model [129, 130]. *E. stiedae* soluble protein was found to stimulate the innate immune response in mice thus, enhancing the antitumor response in a colon cancer model. It promoted DC maturation, activated both CD8 + T cells, and NK cells, and inhibited angiogenesis and metastasis. Both native and recombinant *E*.*tenella* soluble protein extracts exhibited potent immunostimulatory effects, antitumor effects, and the ability to induce IL-12, leading to increased mice survival, reduced tumor size, and high cure rates. *E.tenella* protein shows promise as a stimulator of innate immunity with low toxicity even at high doses [131].

The discovery of potential immunomodulators from parasites could advance cancer immunotherapy. Further research is essential to uncover more parasitic derivatives and develop clinical applications for these immunomodulators. This brings promise to cancer patients, particularly those with advanced disease.

#### Oncolytic agents

The use of live pathogens in cancer treatment has garnered significant interest as a promising therapeutic approach. These microorganisms have shown potential in fighting cancer by directly targeting tumor cells, causing their lysis and stimulating the immune system. Oncolytic organisms induce an immune response against tumors by lysing cancer cells and releasing tumor and pathogen-associated antigens in the tumor microenvironment. This process activates APCs and changes the immunosuppressive tumor microenvironment [132]. These findings highlight the potential of using live microorganisms with lytic activity as part of cancer immunotherapy strategies [133].

Recently, advancements in cancer treatment have shown the use of some viruses and bacteria to target and attack tumors. One such therapy is the oncolytic modified herpes simplex virus (HSV), T-VEC (Imlygic^®^) approved by the FDA for melanoma therapy [134]. Additionally, live bacteria like *Clostridium*,* Salmonella*, *Bifidobacterium*, and *Listeria*, among others have shown potential as effective cancer treatment approaches [135]. However, both therapies face challenges. Viral-based therapy encounters ineffective targeting, tumor microenvironment blocking, and rapid elimination. On the other hand, bacterial therapy has a limited safety frame due to its toxicity [136].

In contrast, biological cancer treatment based on parasites offers a promising alternative to viruses and bacteria. Parasites like *T. gondii*, *Acanthamoeba castellani* (*A.castellani*) and *Neospora caninum* (*N. caninum*) have shown potent oncolytic effects against various types of cancer. For instance, *A. castellanii*, the causative agent of granulomatous amoebic meningoencephalitis and amoebic keratitis effectively destroyed murine melanoma (D5.1G4) and human melanoma (OCM-1) cells through extensive cytolysis. Moreover, its intratumoral injection induced inhibition in tumor size [137]. Similarly, *T. gondii* effectively destroyed Her2/Neu-expressing breast cancer cells (TUBO cells) [138] as well as fibrosarcoma WEHI-164 cells [139], human glioma U373MG 281 cells [140], Merkel cell carcinoma cells, and murine thymoma cancer cells (cell line EG7) [141]. However, the use of live virulent parasites poses potential risks due to the diseases they cause, which may outweigh the benefits.

Therefore, the use of the non-pathogenic parasite *N. caninum*, a livestock parasite that is closely related to *T. gondii*, could be a compelling candidate compared to bacteria, viruses, and other parasites explored in cancer treatment. *N. caninum* is non-pathogenic in humans and has not been detected in human tissues. Neosporosis is not considered a zoonotic disease. *N. caninum* belongs to the phylum Apicomplexa, which possesses an apical complex that enables it to penetrate various cell membranes, including cancer cells, despite the absence of specific host cell receptors. Importantly, it does not integrate into the host cell’s genome, eliminating mutagenic risks [141]. Pre-clinical studies have shown that *N. caninum* can invade human mammary cancer cells (MCF-7 cell line) in vitro, resulting in their lysis [142]. It also induced inhibition of human Merkel cell carcinoma in a mouse model [141] and significantly reduced B16F10 melanoma growth in a C57BL/6 murine model by direct lysis of cancerous cells and induction of strong immune responses [143]. This was evidenced by the increased presence of macrophages and CD8^+^ T cells, along with elevated mRNA expression levels of IL-2, IL-12, IL-10, TNF-α, IFN-γ, and programmed death ligand-1 (PD-L1) in the site of the tumor. Additionally, IFN-γ and IL-12 levels were increased in the spleen of mice with tumors [143].

Similarly, another study demonstrated the oncolytic and immunostimulatory activity of *N.caninum* on EG7 thymoma in syngeneic mice, leading to a significant reduction of both early and established tumor growth and causing their complete elimination when administered via either subcutaneous or intramural injection. The regression of tumors caused by distant *Neospora* administration indicates a protective effect against distant diseases or metastasis [141]. *N.caninum* can reactivate suppressed immune cells at the tumor microenvironment and stimulate the systemic immune system, leading to a long-term antitumor immune response. Additionally, *N. caninum* naturally clears from the tissues contrary to *T. gondii*, which, though induced similar antitumor activity against EG7 thymoma in syngeneic mice, persists in the tumor for a long duration, showing high multiplication and persistence abilities and posing a risk of inducing infection. Furthermore, altering *N. caninum* to produce the IL-15-IL15Ra complex in the tumor microenvironment enhances its immunological characteristics [141].

These findings indicate that *N.caninum* may serve as a promising and safe oncolytic and immunotherapeutic agent for treating human cancer. Engineered strains of *N.caninum* could potentially secrete antitumor agents or express proteins on their surfaces, broadening the range of potential medical use of *N. caninum* in various cancer treatments.

#### Nuclear factor κB (NF-κB) inhibitors

In the past decade, advancements in cancer immunotherapy have resulted in the development of many agents targeting immune checkpoints, such as inhibitory receptors on T cells, like CTLA4 and PD-1 among others. These receptors are vital in regulating the immune response and blocking them can activate the immune system to target cancer cells and prevent tumors from escaping immune detection. Despite the effectiveness of immune checkpoint inhibitors (ICIs), some patients do not respond to them and there may be adverse inflammatory reactions similar to autoimmune diseases, promoting research into combination therapies for cancer treatment [144].

The connection between NF-κB signaling and immune checkpoints has led to investigations into combining NF-κB inhibitors with ICIs to enhance patient response rates. NF-κB is a set of transcription factors that control various biological processes, including cancer-related mechanisms, like abnormal cell growth, survival, and immune evasion. Targeting specific subunits of NF-κB could improve the efficacy of cancer treatments and overcome resistance to traditional therapies like radiotherapy and chemotherapy [145]. To reduce the systemic harmfulness of anti-NF-κB, new approaches aim to selectively inhibit NF-κB subunits in target cells involved in tumor progression. By developing these drugs, researchers hope to improve the efficacy of anti-cancer therapies, including ICIs [146].

Interestingly, parasites have shown potential in the development of novel NF-κB inhibitors. Studies have demonstrated that proteins from *Plasmodium and T. gondii* can inhibit NF-κB, a key player in cancer treatment. For example,, *Plasmodium* circumsporozoite protein, the predominant protein of the sporozoite stage of the malaria parasite, has demonstrated antineoplastic activity against both human lung cancer A549 and colon cancer SW480 cell lines [147, 148]. Additionally, the dense granule protein of *T.gondii* (GRA16) has shown an antineoplastic effect on non-small-cell lung carcinoma in a mouse model [149]. Excretory-secretory products from parasites like *S. equina*,* and T. vaginalis* have also shown promise in reducing NF-κB expression [75, 82, 83]. Further investigation is necessary to explore the potential of parasite proteins as innovative NF-κB inhibitors.

### Targeted therapy

Targeted cancer therapies specifically target and attack cancer cells by acting on possible genetic alteration and unusual proteins that promote cancer growth and spread. Unlike chemotherapy, which affects all rapidly proliferating cells, targeted therapy drugs only target the abnormal proteins [150]. There are two groups of targeted therapy: small-molecule inhibitors and monoclonal antibodies. The later targets specific proteins, such as receptors, on cancerous cells or in the tumor environment. They can block molecules that promote angiogenesis or cancer cell growth, preventing cell proliferation. Monoclonal antibodies can also be combined to toxic substances, like radionuclides or chemotherapy, to deliver treatment directly to cancerous cells. Small-molecule drugs are the other type of targeted therapy that can penetrate cell surfaces to reach intracellular targets and slow growth or induce cell death [151].

Research has demonstrated the potential of certain parasite proteins, such as *E.granulosus* KI-1 (*Eg*KI-1) and refined malaria protein (rVAR2) for targeted cancer therapy. rVAR2 specifically binds strongly to chondroitin sulfate on various cancer cell lines, making it a promising candidate for delivering anti-cancer drugs. When conjugated with a hemiasterlin analog (KT886) to form VDC886, it exhibited strong in vitro cytotoxicity against 33 cancerous cell lines which include melanoma (C3 human and B16 murine cell lines), glioma, human mammary cancer (4T1 and MDA-MB-231 cancerous cell lines), lymphoma (MyLa2059 T-cell line), and human colon cancer cell line (Colo 205) at inhibitory concentrations 50 (IC50) between 0.2 pM to 30 nM. Moreover, VDC886 was tested in a murine model of bone metastatic breast cancer (4T1) and was found to increase the survival time with of mice without any observed metastasis, in contrast to control mice that died from metastatic disease. VDC886 also demonstrated inhibition of non-Hodgkin’s lymphoma (Karpas 299) growth in a mouse model [152].

Additionally, the refined malaria protein rVAR2-drug conjugates, recombinant rVAR2-DT fusion protein, merging the cytotoxic domain of Diphtheria toxin (DT388) with rVAR2, and VDC886 exhibited strong cytotoxicity against human prostate cancer cells (PC-3). They successfully suppressed the proliferation of metastatic castration-resistant prostate cancer cell line, PC-3, in a murine xenograft tumor model. Notably, these conjugates were deemed safe, as they did not induce any toxicity in liver or kidney tissues in the animal model [152].

Furthermore, malaria-mimicking erythrocyte nanoparticles (MMENPs), encased with recombinant VAR2 CSA (rVAR2) and packed with the anticancer drug docetaxel (DTX), have shown promising results in targeting and killing melanoma cells both in vitro and *in vivo.* In a melanoma animal model, these nanoparticles demonstrated significant therapeutic efficacy [153]. Another study introduced a recombinant VAR2CSA peptide (rVAR2)-modified activated platelet-mimicking nanoparticles (rVAR2-PM/PLGA-ss-HA) by encasing disulfide-containing PLGA nanoparticles (PLGA-ss-HA) with an activated platelet membrane and packed them with DTX. These nanoparticles showed a synergistic, antineoplastic activity on primary and lung metastasis in a murine melanoma model (B16F10), reducing tumor volume and weight, decreasing lung metastasis nodules, and inducing tumor cell apoptosis. The targeted delivery of DTX to tumor cells was facilitated by chondroitin sulfate receptors on the tumor cell surface and the activated platelet membrane. This study indicates that utilizing rVAR2-decorated activated platelet-targeting nanoparticles with organized drug delivery may serve as an appealing approach for the treatment of primary and metastatic cancer [154].

The helminthic parasite protein *E.granulosus* KI-1 (*Eg*KI-1), a protein with a molecular weight of less than 10 kDa, has the ability to enter tumor tissues to target intracellular sites to effectively interact with and eliminate cancer cells. *EgKI-1* is a member of the Kunitz-type protease inhibitor family, which is important in cancer therapy due to its role in carcinogenesis and cancer progression [44]. *Eg*KI-1 is highly expressed in the oncosphere of *E. granulosus* and acts as a potent inhibitor of neutrophil elastase and chemotaxis [155]. It has been given an international patent publication [156]. Research has demonstrated that EgKI-1 can enter tumors and efficiently eliminate cancer cells. Recombinant EgKI-1 inhibits the in vitro proliferation and migration of mammary cancer cells (MDA-MB-231, MCF-7, and T47D cell lines) and melanoma cells (CJM) in a concentration-dependent manner by interfering with the cell cycle and triggering apoptosis. Additionally, in a triple-negative breast cancer (MDA-MB-231) murine model, treatment with r*Eg*KI-1 resulted in a significant decrease in tumor size [44].

These results indicate that proteins derived from parasites could have the potential to be promising molecules for targeted cancer therapy, ultimately aiming to selectively kill cancerous cells while sparing normal cells. This discovery highlights the promise of exploring parasites further for the development of innovative treatments that could enhance patients’ outcomes and their quality of life.

### Gene therapy

Gene therapy involves introducing therapeutic genes in cancerous cells to induce cell death or inhibit the growth of cancer. This genetic material which can be either DNA or RNA, is delivered using a vector that helps transfer it to the target cell. Over the years, gene therapy has made significant advancements in the treatment of cancer, with some drugs being approved and others still undergoing trials. Gene therapy is generally considered to be safer and has more tolerated side effects compared to traditional chemotherapy for cancer treatment. Different gene therapy strategies for cancer include suicide gene therapy, activation of tumor suppressor genes, inhibition of oncogene activation, immunotherapy, and antiangiogenic gene therapy [157].

Research studies have shown that parasites can participate in cancer gene therapy through various strategies. As a suicide gene therapy approach, researchers transferred genes encoding the hypoxanthine-guanine phosphoribosyltransferase (HGPRT) enzyme from the protozoan parasite *Trypanosoma brucei (T. brucei)* to cancer cells. This enzyme can more efficiently convert the purine analog, allopurinol, into nucleotides in comparison to the human counterpart. The study developed a new suicide gene/prodrug system that demonstrated specific cancerous cells engineered to express *Tb*HGPRT that could convert the prodrug allopurinol to cytotoxic metabolites, leading to selective apoptosis in certain cancer lines. The treatment was effective in a murine lung cancer cell line (M27), several human non-small cell lung cancer cell lines (H460, A549, H520, H661), and many non-small cell lung cancer cell lines. However, it did not affect other cancer cell lines such as mouse mammary tumor (DA3), bronchioalveolar carcinoma (H322), human hepatocellular carcinoma (HepG2), human breast carcinoma (ZR75, MCF7, MDA231), and neuroblastoma (IMR32). The reasons for the differences in effectiveness in different cancer cell lines remain unexplained as neither the cellular accumulation of the prodrug nor the expression of the *Tb*HGPRT gene can justify the differences in cytotoxicity observed among the tested cell lines [158].

Furthermore, research has shown that dysregulated expression of specific miRNAs is associated with the onset of different diseases, like cancer [159]. Studies have revealed that *S. japonicum* miRNA is detected in the hepatocytes of *S. japonicum-*infected mice, potentially boosting the resistance of the host to liver cancer. Experiments have demonstrated that *Sja-*miR-71a, *Sja-* miRNA-7-5p, *Sja*-miR-61, and *Sja*-miR-3096 mimics significantly inhibited the migration of various liver cancer cell lines (human hepatoma (HepG2), human HCC (SMMC-7721), and mouse hepatoma (Hepa1-6) [160–163]. In a xenograft murine model, animals injected with miRNA mimics transfected cells showed reduced tumor growth, volume, and weight by targeting specific tumor genes. For example, *Sja*- miRNA-7-5p decreases the expression of the S-phase kinase-associated protein 2 gene [161], *Sja*-miR-61 suppresses angiogenesis by targeting the PGAM1 gene [160], *Sja*-miR-3096 targets the phosphoinositide 3-kinase class II alpha (PIK3C2 A) gene [163], and *Sja*-miR-71a targets the FZD4 gene [162]. This suggests that exogenous miRNAs like *Sja*-miR hold promise for cancer gene therapy.

Additionally, the muscle larvae proteins of *T.spiralis* caused significant changes in the transcriptome of A549 lung cancer cells, affecting 2,860 genes expression. Among these genes, 1,634 were up-regulated while 1,226 were down-regulated, impacting pathways related to cancer and metabolic processes. Notably, glycolysis-related genes were down-regulated, potentially harming A549 lung cancer cells [57]. Other studies have also indicated that apoptosis-related genes in *T.spiralis* have a role in suppressing the growth of cancer. Research has shown that *T. spiralis* crude extract containing components from adult worms and newborn larvae suppressed the proliferation of various cancer cell lines in vitro, including murine ascitic hepatoma (H22), murine forestomach carcinoma (MFC), human hepatoma (H7402), murine sarcoma (S180), and human chronic myeloid leukemia (K562), and significantly promoted regression of MFC, H22, and S180 in animal models [164]. Additionally, excretory-secretory proteins of *T. spiralis* muscle larvae induced apoptosis in non-small-cell lung cancer cells (A549), small-cell lung cancer cells (H446 SCLC), and melanoma cells by stimulating the mitochondrial apoptotic pathway. This was demonstrated by the down-regulation of Bcl-2 and Livin genes and up-regulation of sit-in, Apaf-1, caspase-9, and caspase-3 genes [56, 58, 165]. Furthermore, *T. spiralis* contains ribosomal proteins, S24 and S24e, which are overexpressed in various cancers like colorectal, gastric, esophageal [61]. These proteins play roles in cell proliferation regulation, DNA repair, and cell differentiation. Interestingly, a recent study demonstrated that *Trichinella* iRNA inhibited the growth of myeloma tumors (SP2/0) in BALB/c mice [166].

Furthermore, the synthesized peptide fraction derived from *T. canis* excretory-secretory Troponin protein, exhibited antineoplastic activity on colon cancer cell lines (Caco2 and HT-29). This activity was achieved by modulating genes expression (APAF1, Mcl-2, VEGF, cyclin-D1, ZEB1 and caspase-3) associated with cancer development [167].

Also, the dense granular protein 16 (*Tg*GRA16), derived from *T.gondii* shows potential for gene therapy. In an in vitro study, *Tg*GRA16 was found to inhibit telomerase activity, leading to apoptosis in human colorectal cancer HCT116 cells by reducing the expression of human telomerase reverse transcriptase which led to telomere shortening due to telomerase inactivation, which was facilitated by the activation of tension homolog and the tumor suppressor phosphatase [168].

Additionally, exosomes from parasites obtained from the culture supernatants of *T.gondii (*Me49 strain) infected DCs have shown antitumor effects in a CRC murine model. These exosomes suppressed tumor growth and the polarization of macrophages to the M2 phenotype, as well as regulated the expression of suppressor of cytokine signaling 1 (SOCS1) by delivering miR-155-5p. They also reduced the percentage of monocytic myeloid-derived suppressor cells (M-MDSCs, CD11b^+^Ly6C^+^) and polymorphonuclear granulocytic bone marrow-derived suppressor cells (G-MDSCs, CD11b^+^Ly6G^+^), while increasing DCs (CD45^+^CD11c^+^). Additionally, they decreased levels of granulocyte-macrophage colony-stimulating factor and IL-6 by suppressing the signal transducer and activator of the transcription signaling pathway. This suggests a potential new cancer treatment strategy by converting a “cold” tumor into a “hot” tumor, which could enhance the delivery of drugs and antibodies in CRC gene immunotherapy [169].

Another study showed that infecting mice bearing LLC with non-lethal *P. yoelii* inhibited tumor growth by upregulating F630028O10Rik (F63), a newly discovered long non-coding RNA. Treatment of mice with *P.yoell*i-infected red blood cells showed significantly higher levels of F63 in their tumor tissues in comparison to the control group. F63 was found to suppress VEGF secretion in tumors and inhibit various processes involved in endothelial cell functions such as clone formation, invasion, migration, and tube formation. These findings suggest that F63 can effectively inhibit tumor growth and progression by regulating angiogenesis, making it a potential candidate for antiangiogenic gene therapy in lung cancer [170].

Hence, it is noteworthy to thoroughly explore parasites and their derived molecules to potentially develop novel gene therapeutics that may help in managing cancer as a controllable disease.

### Synergistic combination therapy

Combination therapy is a master strategy for the treatment of cancer engaging multiple anti-cancerous drugs to enhance effectiveness and combat drug resistance. This strategy may exhibit antagonism, synergy, or additivity [171]. A recent research analyzing phase III trial data found that most approved drug combinations in oncology show additive, rather than synergistic, clinical efficacy. Synergistic effects between drugs are rare and have led to the need for innovative approaches to optimize therapy regimens [172].

Recent research in the era of parasites’ antineoplastic activities has shown promising synergistic effects with conventional cancer treatments like chemotherapy and radiation therapy. Using a murine melanoma model via B16-F10 cells, the synthetic peptide GK1 derived from *T. crassiceps* cysticerci demonstrated anti-melanoma activity. Treatment with GK1 significantly prolonged melanoma-bearing mice’s survival time, slowed down the growth of the tumor and promoted necrosis within the tumor suggesting its potential anticancerous activity via promoting pro-inflammatory cytokines [114]. Upon the combination of GK1 with anti-programmed death ligand-1, GK1 showed even greater survival benefits when compared to either treatment. The combination therapy reduced serum cytokine levels as IL-4–6, IL-10, and TNF-α indicating the synergistic effects of GK1 [173]. Additionally, an in vitro study demonstrated the effective value of combining recombinant *T. solium* calreticulin (rTsCRT) with 5-fluorouracil which resulted in a 60% inhibition of viability in human breast and ovarian cancer cell lines, MCF7 and SKOV3 respectively [174].

Additionally, combined administration of attenuated tachyzoites (RH- ΔGRA17) strain and PD-L1 blockade intratumorally in a mouse colon adenocarcinoma model (MC38) significantly enhanced mice survival, and inhibited tumor growth when compared to individual treatments. Moreover, this combined therapy boosted lymphocyte infiltration and led to the sensitization of both local as well as distant tumors to PD-L1 blockade treatment [175]. Similarly, in a pancreatic ductal adenocarcinoma subcutaneous mouse model (Pan02 tumor-bearing mice), combining attenuated *Toxoplasma* of the NRTUA strain with an anti-PD1 antibody significantly enhanced the antitumor activity, induced a strong immunogenic response, with effectively controlling tumor growth. This combination therapy targeted the myeloid-derived suppressor cells and modified the immunosuppressive tumor microenvironment [176]. Moreover, in a non-small-cell lung carcinoma mouse xenograft model, *T.gondii* derived-dense granule protein 16 (GRA16) has shown anticancer properties by reducing tumor size. It was found to enhance the effect of Irinotecan; an anticancer drug, when Irinotecan and GRA16 were administered together, they synergistically promoted the anticancerous effect of Irinotecan by inducing a cell cycle arrest and inhibiting NF-κB. This combination increased Irinotecan’s efficacy for the treatment of non-small-cell lung carcinoma [149].

In a murine model of LLC, infection of mice with *P. chabaudi* combined with Gemcitabine inhibited tumor growth and metastasis significantly, showing a synergistic effect and prolonging mice survival. This combination therapy may inhibit tumor cell epithelial-mesenchymal transition via blocking CXCR2/TGF-β-mediated PI3K/Akt/GSK-3β/Snail signaling pathway, suggesting potential for clinical trials in lung cancer patients [177]. Similarly, immunotherapy with *P. yoelii* combined with radiotherapy in both mice models of glioma (GL261) and non-small LLC demonstrated synergistic antitumor effects by modulating immune cell populations, up-regulation of effector CD8 + T cells expressing perforin, and down-regulation of the myeloid-derived suppressor cell population. This combination therapy effectively prolonged the survival of mice, slowed tumor growth, and successfully achieved a cure rate of approximately 70% in glioma models, suggesting potential for clinical trials in glioma treatment [178].

These findings would enhance the effectiveness of cancer combination therapy and ultimately improve patient outcomes.

### Combating resistance to Anti-Cancer drugs

Drug resistance in cancer occurs when cancer cells become tolerant to treatment, whether it is traditional chemotherapy or newer targeted therapies. This resistance, whether present before treatment (intrinsic) or developed during treatment (acquired), is a major factor in cancer relapse and mortality. Various factors, including genetic and epigenetic mutations with increased drug efflux among others, contribute to drug resistance in cancer [179]. The complexity and adaptability of cancer cells make overcoming drug resistance a significant challenge.

Understanding the mechanisms behind drug resistance is crucial for improving cancer treatment outcomes [180]. Increased efflux of anticancerous drugs is a mechanism by which drug resistance is achieved leading to lower drug levels within the cells [181]. Interestingly, infection or exposure of cancer cells to *T. gondii* or its lysate antigen can reduce drug resistance in multi-drug resistant gastric cancer and murine lymphoma cancer cell lines; as Mdr L 5718 and Par 5718 via increasing the accumulation of drugs through interference with the drug efflux pump which is dependent on ATP [182].

This innovative approach will potentially overcome the developed resistance against anti-cancerous drugs, enhancing their effectiveness and improving patient outcomes. Further in-depth research is warranted to explore its benefits.

## Concluding remarks

Cancer is considered a deleterious disease leading to high rates of human morbidity and mortality, with millions of new cases diagnosed annually. Therefore, cancer research has focused on developing innovative treatments such as precision medicine, immunotherapy, and targeted therapy. Immunotherapy and targeted therapy are rapidly advancing treatments that are widely considered groundbreaking in cancer therapy and have significantly improved therapeutic strategies. There is growing interest in using parasites for cancer immunotherapy, utilizing parasites, their derived molecules, and antisera. These approaches have shown promise in cancer treatment by offering several mechanisms to fight cancer. These include direct promotion of apoptosis and cell cycle arrest. Additionally, the presence of shared antigens between parasites and cancer cells is believed to play a key role in parasite-based cancer therapy. Parasites and their derivatives can alter the tumor microenvironment from immunosuppressive to immunostimulatory, which is a crucial anti-cancer mechanism. Parasite-based therapies target angiogenesis in the tumor microenvironment by inhibiting vascular development through the suppression of VEGF. Interestingly, via an indirect mechanism, VEGF has an immune-suppressor activity. Consequently, interfering with angiogenesis stimulates the immune system. These findings have sparked interest in exploring this novel biological immunotherapeutic approach for combating cancer. Parasites that cause little to no harm are appropriate candidates for this purpose. However, a more ideal approach would involve discovering molecules from parasites that have anti-cancer properties, making them more suitable for human use.This innovative approach may pave the way for a new wave of new and effective anti-cancer therapies. By exploring the molecular mimicry between wide range of parasites and different types of cancer, as well as identifying common antigens and their corresponding antibodies, characterizing them, and assessing their safety and efficacy, novel immunotherapies such as potent cancer vaccine candidates, novel parasitic adjuvant platforms, and novel approach for developing therapeutic antibodies for targeted cancer immunotherapy could be discovered. Additionally, the identification of immune system modulators and innovative NF-κB inhibitors from parasites could advance cancer immunotherapy. Further research on *Neospora caninum* and non -pathogenic intracellular parasites could lead to the discovery of safe and effective parasitic oncolytic and immunotherapeutic agents. Investigating the use of engineered non-pathogenic oncolytic parasite strains to deliver anti-tumor agents could expand the potential medical application of these oncolytic agents in various cancer treatments. Certain parasite proteins could hold promise as targeted cancer therapy molecules. Furthermore, parasites could provide personalized treatment options and gene therapy by creating new gene therapeutics to help manage cancer as a controllable disease. They could also be used in combination with other cancer immunotherapeutic agents to enhance effectiveness and potentially overcome drug resistance, improving the outcomes of cancer combination therapy and improving patient prognosis. Thus, parasites offer a promising avenue for introducing a new generation of cutting-edge anti-cancer treatment options, suggesting the need to further investigate their antineoplastic properties, develop clinical applications, and advance promising results to clinical trials. This could renew hope and offer potential new options for cancer patients..

## Data Availability

The dataset(s) supporting the conclusions of this article are included within the article.
